# Inflammation Mediates Body Weight and Ageing Effects on Psychomotor Slowing

**DOI:** 10.1038/s41598-019-52062-3

**Published:** 2019-10-31

**Authors:** Leonie J. T. Balter, Suzanne Higgs, Sarah Aldred, Jos A. Bosch, Jane E. Raymond

**Affiliations:** 1 School of Psychology, University of Birmingham, Birmingham, B15 2TT UK; 2 School of Sport, Exercise, and Rehabilitation Sciences, University of Birmingham, Birmingham, B15 2TT UK; 3 Psychology Department, Clinical Psychology, University of Amsterdam, Amsterdam, 1018 WT The Netherlands

**Keywords:** Neuroimmunology, Human behaviour

## Abstract

Inflammation (immune system activation) affects neuronal function and may have consequences for the efficiency and speed of functional brain processes. Indeed, unusually slow psychomotor speed, a measure predictive of behavioural performance and health outcomes, is found with obesity and ageing, two conditions also associated with chronic inflammation. Yet whether inflammation is the mediating factor remains unclear. Here, we assessed inflammation by indexing interleukin-6 level in blood and measured psychomotor speed as well as indices of selective visual attention in young (mean = 26 years) or old (mean = 71 years) adults (*N* = 83) who were either lean or currently significantly overweight (mean body mass index = 22.4 and 33.8, respectively). Inflammation was positively and significantly correlated with psychomotor speed, age, and body mass index but not with attention measures. Using mediation analyses we show for the first time that inflammation fully accounts for the significant psychomotor slowing found in those with high BMI. Moreover, we further show that age-related psychomotor slowing is partially mediated by inflammation. These findings support the proposal that reducing inflammation may mitigate weight- and age-related cognitive decline and thereby improve performance on daily tasks and health outcomes more generally.

## Introduction

Inflammation, a biological process that results from immune system activation, is a well-established characteristic of advanced age^1,2^ and obesity^3^. At the cellular level, inflammation is known to cause neuronal dysfunction via alterations to microglia^4–6^, but how such alterations affect overt brain function remains less well studied. Nevertheless, emerging evidence tentatively links inflammatory states with subtle cognitive deficits, including slowed psychomotor speed, i.e., the minimum time required to make accurate, well-learned motor responses to obvious sensory stimuli^7^, slowed decision processes^8^, and memory deficits^9,10^, but see^11^; also^12^. Importantly, many of these same behavioural deficits are also found in individuals with obesity^13,14^ as well as in those in advanced age^15^. Taken together, the parallel relationships of both age and weight with cognitive function and inflammation raise the possibility that inflammation may be the underlying mechanism by which advanced age and excessive body weight lead to suboptimal cognitive capacity. Yet no study to date has examined how inflammation, age, and obesity interact to determine human cognitive performance using a participant sample that varies substantially on these dimensions. Not only does it remain unclear whether age and body weight are additive or multiplicative in their impact on performance, the hypothesis that inflammation may be the mediating mechanism remains untested.

**Figure 1:**
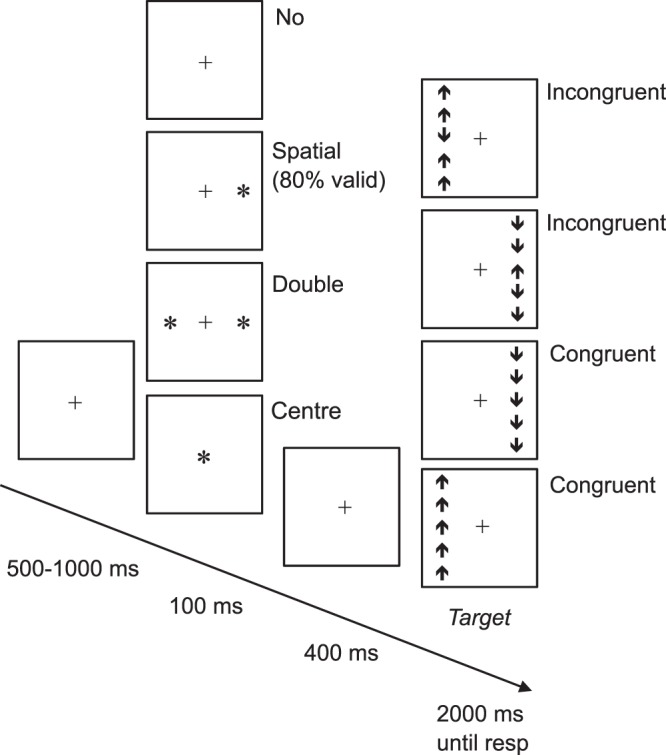
Experimental task design. The sequence of displays presented in each trial of the ANT task. The cue display had four equally likely conditions: no cue, spatial cue (80% valid), double cue, and centre cue. The target array comprised a central arrow and four identical flankers that were either congruent or incongruent with the target arrow. The task was to report the direction (up, down) of the centre target arrow as quickly and accurately as possible.

A fundamental brain function that underpins many everyday activities is the capacity to respond rapidly and appropriately to new or changing visual information in a manner consistent with ongoing and future goals^16^. This requires appropriate selection of relevant information from the everchanging complex visual sensory environment, as well as the capacity to quickly activate appropriate motor responses as soon as cognitive decisions, e.g., object identification, have been reached. The former set of processes are known as selective visual attention^17^ and the latter as psychomotor speed (e.g.^18^). A typical example of the importance of these functions is fast braking in the face of a sudden, unexpected road obstacle. Selective attention directs processing towards the obstacle; psychomotor speed reflects the time needed to press the brake pedal. To efficiently select relevant information (targets) from a scene, discrete cognitive sub-functions subserved by different but overlapping brain networks are used^19–22^. One network function, Alerting, promotes selection by reacting to sensory cues that signal *when* a target might appear; another, Orienting, reacts to cues signalling *where* a target might appear; and a third, Executive Control, promotes high level processing of task relevant information and suppresses processing of concurrent distracting information. Such functions can be assessed using the Attention Network Test (ANT) which requires participants to respond as quickly and accurately as possible to a series of simple computer-based trials, each comprised of a cue display that is quickly followed by a target array. See Fig. 1. The speed and accuracy of key press responses to targets are measured and compared across different cue-target conditions^20–22^. In addition to comprehensively assessing attention, this test provides a robust measure of psychomotor speed (overall mean response time, RT, across all cue conditions). Although psychomotor speed in everyday scenarios depends on the nature of the information being presented and the complexity of the action decisions required^23^, its measurement when based on simple manual motor responses, such as required in the ANT, is nevertheless predictive of a range of performance outcomes, e.g., driving^24^, health outcomes, e.g., response to depression treatment^25^, and even mortality risk^26^.

Decline in attention and slowing of psychomotor speed has long been linked to ageing^27^ and has been well-studied using the ANT^21,28–31^. Such deficits are thought to contribute to frailty, risk of falling, depression, and poor health^32–35^. Several studies report weaker alerting benefits and/or diminished distractor suppression, an index of executive control^29–32^. However, after adjusting for the effects of generalised psychomotor slowing^28^, age-related attentional deficits disappear or become weaker^28,29,36^. Hence, ageing may have only modest effects on attention beyond generalised slowing, an issue we probe here.

The notion that age-related cognitive decline may be mediated by inflammation^37,38^ has received modest attention. Lin *et al*.^8^ for lean adults, inflammation only partially mediated the association between age and complex cognitive processing speed and did not mediate links between age and short-term memory. Other studies show clear negative correlations between complex cognitive performance and inflammation in midlife^9,39^ and in older participants^37^, but did not conduct mediation analyses nor consider the role of body weight. Indeed, recent evidence provides strong support for the contention that age-related cognitive decline may be accelerated by excessive body weight, causing pre-senescent cognitive deficits^38,40^. A high BMI across the life span is associated with lower performance across several cognitive domains that are characteristically degraded in ageing, including verbal memory^41,42^, executive functioning^43–45^ and, complex visual-motor coordination^46^. However, studies on the effects of excessive body weight on psychomotor slowing and selective visual attention are less clear. Some report weight-related deficits^42,47,48^, others find none^40,49,50^; one reported better performance with higher BMI^51^. Complicating this picture is evidence that depression and poor cardiovascular health, conditions that are co-morbid with obesity, may themselves contribute to reduced cognitive function^52,53^. Indeed, Prickett *et al*.^14^, reviewed findings that linked obesity with cognitive deficit and concluded that evidence was equivocal as to whether cognitive problems stem from obesity or from other co-morbid health and demographic factors. Nevertheless, using a very large sample of exclusively older adults and adjusting for other co-morbid factors in a mediation analysis, Bourassa & Sbarra^12^ report support for a causal role of inflammation in weight-associated deficits on a delayed recall measure of memory.

Support for the possibility that inflammation may be a driving mechanism of psychomotor slowing is found in studies that acutely or experimentally induced inflammation and measured cognitive function with and without inflammation. For example, the common cold, a condition that produces an acute inflammatory response, leads to psychomotor slowing^54,55^ and translates to slower responses in a simulated driving task^56,57^. Experimentally induced inflammation via administration of immune-activating agents, such as bacterial endotoxin or vaccination, also causes psychomotor slowing^7^, memory deficits^10^ and alterations of social cognition^58,59^. However, the extant literature using experimental induction of inflammation so far provides little evidence of degraded selective attention^60^ (reviewed in^61^), suggesting the possibility of domain-specificity in the inflammation-cognition link.

Taken together these findings support the notion that psychomotor slowing, shown clearly to be associated with advanced age and possibly with excessive body weight, may be mediated by chronic inflammation. The aim of the current study was to test this hypothesis by determining whether inflammation (as indexed by a generic marker of systemic inflammation; IL-6) is a mediator of age- and BMI-related psychomotor slowing. Additionally, we aimed to determine whether age and body weight are independent^50,62^ or interactive^51^ predictors of psychomotor speed and visual attention. We predicted that individuals with high BMI and advanced age would show higher levels of inflammation and slower psychomotor speed compared to their leaner and younger counterparts. The association between BMI, age, and psychomotor speed was expected to be at least partially mediated by inflammation. Furthermore, attentional processing was not expected to be reduced by either older age or high BMI when psychomotor speed was taken into account (e.g.^29^).

## Results

### Inflammation

**Figure 2:**
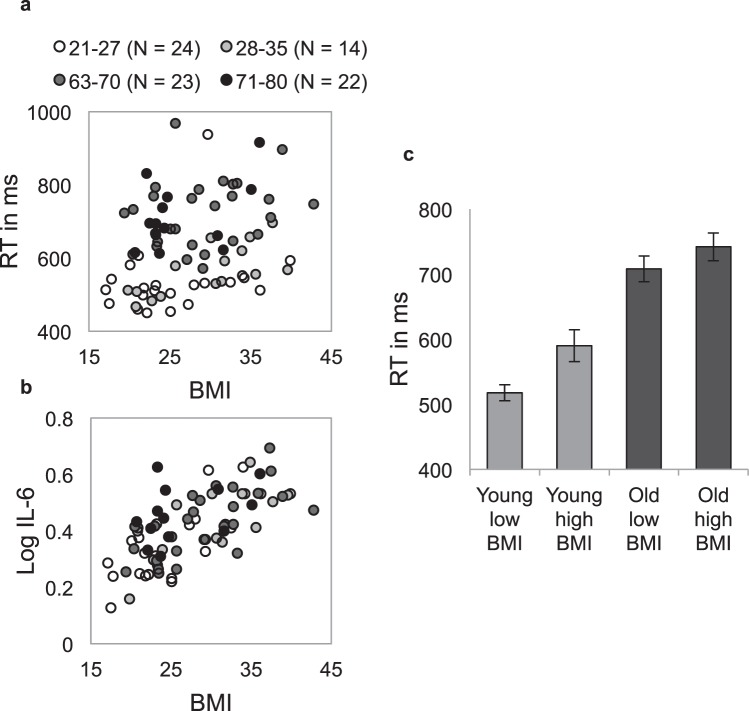
Correlations of BMI with IL-6 and psychomotor speed (RT), and RT differences between groups. (**a**) Individual Log IL-6 level or (**b**) psychomotor speed plotted as a function of BMI. Each dot represents a single participant and dot gray scale reflects the participant’s age. Log IL-6 significantly correlated with BMI (*r*_*S*_(81) = 0.655, *p* < 0.001) and Age (*r*_*S*_(81) = 0.320, *p* = 0.003). Overall RT significantly correlated with BMI (*r*_*S*_(81) = 0.334, *p* = 0.002) and Age (*r*_*S*_(81) = 0.659, *p* < 0.001) (**c**). Mean psychomotor speed for each group. Error bars represent standard error of the mean (s.e.).

The study was conducted on four participant groups who differed in age (young, old) and BMI (low, high), but not in education level. The sample characteristics are presented in Table 1. To assess the effects of age and BMI on inflammation, an analysis of variance (ANOVA) was conducted on IL-6. Confirming expectations, IL-6 levels were higher in the high versus low BMI groups (*F*(1, 79) = 49.25, *p < *0.001, η_p_^2^ = 0.37), and also higher in the old versus young groups (*F*(1, 79) = 4.55, *p* = 0.036, η_p_^2^ = 0.03). The interaction of age and BMI was non-significant (*F*(1, 79) = 1.62, *p* = 0.207, η_p_^2^ = 0.01). Mutual adjustment (i.e., BMI adjusted for age (*F*(1, 80) = 49.00, *p < *0.001, η_p_^2^ = 0.38), and age adjusted for BMI (*F*(1, 80) = 4.06, *p* = 0.047, η_p_^2^ = 0.05) yielded a comparable pattern of results. IL-6 significantly correlated with BMI (*r*_*S*_(81) = 0.655, *p* < 0.001) and with age (*r*_*S*_(81) = 0.320, *p* = 0.003). See Fig. 2a.

**Table 1:** Descriptive statistics of participant characteristics.

Characteristic	Young Low BMI	Young High BMI	Old Low BMI	Old High BMI	Main effects	
					Age	BMI
N	20	18	20	25		
Age (years)						
Mean	24.9	27.3	72.2	69.5	Age***	
Range	21–32	21–35	66–79	63–76		
Sex (% Female)	45	39	40	40		
IL-6 (pg/ml)	1.04 (0.44)	2.13 (0.67)	1.44 (0.61)	2.32 (1.01)	Age*	BMI***
Range	0.3–2.1	1.1–6.0	0.8–3.2	1.1–6.0		
Weight Status						
BMI (kg/m^2^)	21.7 (2.5)	33.2 (3.7)	23.1 (1.8)	32.5 (3.9)		BMI***
BMI when young (21–35) (kg/m^2^)			21.8 (1.6)	25.9 (4.5)		
Body fat %						
Females	27.9 (3.9)	45.3 (4.8)	33.6 (5.8)	44.9 (4.8)		BMI***
Males	15.7 (4.4)	28.9 (3.8)	22.4 (5.0)	31.6 (5.1)		BMI***
Time of day tested (start time)	11h34 (1h45)	11h16 (1h27)	11h31 (1h51)	11h22 (1h15)		
Education level %						
Higher	100	72	75	72		
Middle	0	17	25	28		
Lower	0	11	0	0		
Self-reported health (scale 1–10)	8.4 (0.9)	7.6 (1.4)	8.7 (0.9)	8.0 (1.2)		BMI***
Illness symptoms (SCQ)	0.7 (1.0)	1.3 (2.0)	2.7 (2.0)	4.9 (3.2)	Age***	BMI**
History of illness symptoms (SCQ)			2 (1.7)	2.6 (3.2)		
Sum of medication	0.1 (0.3)	0 (0)	0.9 (1.4)	1.7 (1.6)	Age**	
Range	0–1	0–1	0–4	0–5		
Occupation status^%						
Employed	10	22	10	0		
Student	90	50	0	0	Age***	BMI*
Retired	0	0	85	100	Age***	
Voluntary work	0	0	10	20	Age*	
Unemployed	0	28	5	0		
Smoking %						
Current smoker	0	6	0	0		
Ex-smoker	5	18	25	44	Age*	
Never smoker	95	76	75	56		BMI**
Alcohol intake in units	1.6 (3.3)	1.7 (3.3)	6.3 (4.5)	7.7 (10.2)	Age***	
Emotional health						
Depression	4.7 (6.1)	7.4 (7.5)	1.1 (2.0)	2.8 (3.7)	Age***	
Anxiety	4.7 (5.0)	6.0 (5.8)	0.6 (1.3)	1.5 (2.0)	Age***	
Stress	10.3 (7.5)	10.4 (7.0)	4.8 (4.5)	5 (4.8)	Age***	
Loneliness	37.2 (7.9)	37.4 (7.5)	35.3 (6.0)	36.5 (6.5)		

Numbers in parenthesis indicate s.d.; Statistical significance of main effects are indicated as follows; *p < 0.05, **p < 0.01, ***p < 0.001. All interactions involving Age or BMI group were non-significant; ^Please note that percentage may add up to more than 100% due to multiple answer possibilities.

### Behavioural measures

The ANT task was used to measure psychomotor speed (overall RT computed using all task conditions) and visual attention. Participants were shown one of four warning cues followed by a target arrow flanked by four similar arrows pointing in the same (congruent) or opposite (incongruent) direction as the target arrow. As illustrated in Fig. 1, the participant’s task was to report the direction of the central target as quickly and accurately as possible. The four different warning cue conditions were: (1) no cue, (2) double alerting cue (one cue appearing simultaneously at each possible target location), (3) central alerting cue (appearing in place of the fixation cross), and (4) spatial cue (a single cue appearing at one of the two possible target locations). The spatial cue predicted the target location correctly on 80% of trials (valid trials) and incorrectly on 20% of trials (invalid trials). Differences in RT for the different cue and target-flanker conditions were used to index the efficiency of separate components of attention in each individual. As is conventional^21^, the alerting network was assessed by comparing performance on double-cue versus no-cue trials; the orienting network was assessed by comparing performance on valid spatial cue trials versus double-cue trials; validity effects were indexed by comparing performance on valid versus invalid spatial cue trials; and lastly, the executive control network was assessed by differences in performance for congruent versus incongruent flanker trials, regardless of cue condition.

ANOVA of individual condition mean RTs obtained in the ANT task revealed main effects of Age group (*F*(1, 79) = 68.36, *p* < 0.001, η_p_^2^ = 0.46), BMI group (*F*(1, 79) = 6.62, *p* = 0.012, η_p_^2^ = 0.08), Target Flanker condition (*F*(1, 79) = 89.42, *p* < 0.001, η_p_^2^ = 0.53), and Cue condition,(*F*(4, 316) = 20.08, *p* < 0.001, η_p_^2^ = 0.20). As expected the interaction of Cue X Target Flanker condition was also significant (*F*(4, 316) = 2.69, *p* = 0.031, η_p_^2^ = 0.03), but neither this effect nor any other effect of target flanker or cue condition interacted with Age or BMI group (all *F*’s < 1.2). Critically, Age and BMI group did not significantly interact (*F*(1, 79) = 0.68, *p* = 0.412, η_p_^2^ = 0.01). The group mean RTs and accuracy for each target flanker and warning cue condition for each age and BMI group are shown in Tables 2 and 3.

**Table 2:** Mean RTs (s.e.) as a function of target flanker condition and warning cue condition for each Age and BMI group.

	Target Flanker condition		Warning Cue condition					
	Congruent	Incongruent	No	Centre	Double	Valid	Invalid	Mean-RT
Young Low BMI	490 (11)	550 (14)	524 (12)	519 (11)	523 (14)	502 (12)	552 (17)	518 (12)
Young High BMI	556 (18)	625 (31)	601 (22)	591 (24)	602 (29)	571 (26)	606 (19)	590 (25)
Old Low BMI	678 (15)	740 (29)	708 (17)	705 (25)	723 (20)	693 (19)	734 (23)	708 (20)
Old High BMI	707 (21)	778 (23)	749 (22)	739 (21)	753 (22)	722 (21)	787 (33)	742 (21)

**Table 3:** Mean accuracy scores in % (s.e.) as a function of target flanker condition and warning cue condition for each Age and BMI group.

	Target Flanker condition		Warning Cue condition					
	Congruent	Incongruent	No	Centre	Double	Valid	Invalid	Mean-%
Young Low BMI	98.1 (0.6)	93.8 (1.5)	95.9 (1.4)	95.5 (1.1)	95.6 (1.1)	96.7 (0.9)	94.7 (2.1)	95.9 (1.0)
Young High BMI	98.6 (0.4)	94.8 (1.3)	96.9 (1.0)	96.3 (1.1)	95.8 (1.1)	97.4 (0.6)	96.5 (1.4)	96.7 (0.8)
Old Low BMI	99.0 (0.3)	97.7 (0.8)	98.1 (0.9)	98.0 (0.5)	98.5 (0.6)	98.6 (0.4)	98.1 (0.7)	98.3 (0.4)
Old High BMI	99.0 (0.4)	98.1 (0.6)	98.1 (0.7)	99.0 (0.4)	98.6 (0.5)	98.9 (0.5)	97.5 (1.0)	98.6 (0.4)

### Psychomotor speed (overall RT)

Main effects of Age and BMI describe group effects on psychomotor slowing (overall RT), a point of primary interest for the current study, and therefore merit further examination. Contrasting with some previous studies^40^, we found that individuals with high BMI were 53 ms (s.e. = 20 ms) slower than lean individuals (adjusted for age: *F*(1, 79) = 6.90, *p* = 0.010, η_p_^2^ = 0.08) and that BMI was positively correlated with overall RT (*r*_*S*_(81) = 0.334, *p* = 0.002). See Fig. 2b. Confirming previous reports^27^, psychomotor speed for older adults was on average 169 ms (s.e. = 20 ms) slower than that for young adults (adjusted for weight: *F*(1, 79) = 70.98, *p* < 0.001, η_p_^2^ = 0.47) and age and overall RT were highly correlated (*r*_*S*_(81) = 0.659, *p* < 0.001) (see Fig. 2). Adjustments for relevant demographic and health-related variables listed in Table 1 did not alter any of these results related to psychomotor speed or attention (all *F*’s < 1). Additionally, body fat percentage (BF%) and BMI were highly correlated in the current sample (males r(48) = 0.902, p < 0.001; females r(33) = 0.860, p < 0.001), suggesting that BF% and BMI measure similar aspects of body mass. Moreover, the correlation between BMI and RT (r_s_(81) = 0.334, p = 0.002) and BF% and RT were very similar (r_s_(81) = 0.360, p < 0.001).

As shown in Table 3, accuracy across all groups was high (*M* = 97.7%, s.e. = 0.3%), although accuracy was 1.8 percentage points better for old versus young participants (*F*(1, 78) = 20.52, *p* = 0.002, η_p_^2^ = 0.12), suggesting that speed accuracy trade-offs may have contributed to age-related slowing. Accuracy did not differ between the two BMI groups (*F* < 1).

**Figure 3:**
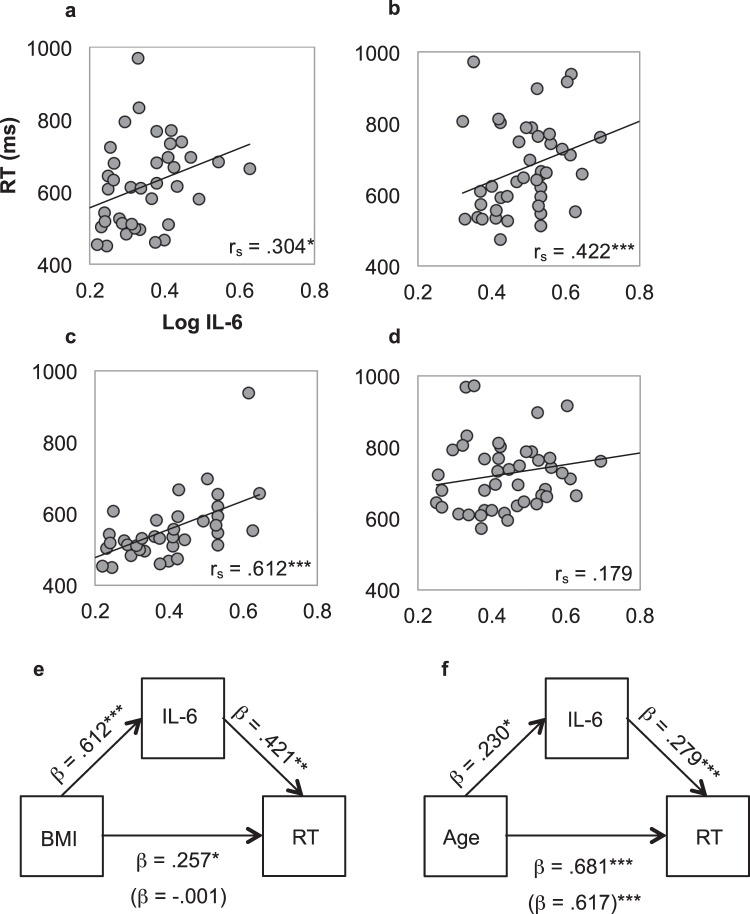
Spearman’s correlations between IL-6 and psychomotor speed (RT), and standardised regression coefficients of the mediation analyses. Spearman’s correlations between IL-6 levels and psychomotor speed (RT) in the low BMI (**a**), high BMI (**b**), young (**c**), and old (**d**) groups. Standardised regression coefficients of the mediation analyses for the BMI and age groups are shown in (**e**,**f**), respectively. Age and BMI group significantly predicted RT and IL-6 level. IL-6 level still significantly predicted RT once the effect of BMI/Age on RT was taken into account. However, the effect between BMI and RT became non-significant after accounting for differences in IL-6, suggesting that IL-6 fully mediates the effect between BMI and RT. The effect between Age and RT became weaker but remained significant after taking into account IL-6, suggesting that IL-6 (only) partly mediates the effect of Age on RT. Indirect effects after taking mediation into account are presented in parentheses. *p < 0.05, **p < 0.01, ***p < 0.001.

As shown in Fig. 3a–d, psychomotor speed was significantly correlated with inflammation for the low (*r*_*S*_(40) = 0.304, *p* = 0.047) and high BMI (*r*_*S*_(43) = 0.422, *p* = 0.007) groups; a similar effect was also found for the young group (*r*_*S*_(38) = 0.612, *p* < 0.001) but was absent in the old group (*r*_*S*_(45) = 0.179, *p* = 0.238). This supports the idea that performance in ageing is affected by multiple factors. To investigate the hypothesis that inflammation mediated the relationship between overall RT and either BMI or age, mediation analyses were performed on overall RT scores. As shown in Fig. 3e, when IL-6 was entered as a mediating variable, BMI group was no longer a significant predictor of RT (β = 0.00, 95% CI = −0.26–0.25) indicating that inflammation level fully mediated the relationship between BMI and RT (β = 0.26, 95% CI = 0.09–0.46). As a further test, individuals were divided into two groups based solely on inflammation level (using a median split), and the group’s overall RTs were compared. The high inflammation group was on average 90 ms (s.e. = 2.7 ms) slower compared to the low inflammation group (*t*(81) = −3.40, *p* = 0.001; *d* = − 0.747, independent 2-tailed).

The mediation analysis also showed a significant indirect effect of inflammation on the association between age and overall RT (β = 0.06, 95% CI = 0.01–0.15). However, age remained a significant predictor of RT after accounting for the mediating effect of IL-6 (β = 0.62, 95% CI = 0.46–0.77), indicating that IL-6 only partially mediated the effect of age on RT (see Fig. 3f). Adjusting for sex, education level, time of day, perceived health status, depression, anxiety, stress, illness symptoms, smoking status, and alcohol intake and repeating the above mediation analyses using hierarchical regression analysis did not significantly alter results (see Supplementary information Tables S1 and S2).

### Visual attention

Results of the main ANOVA showed only main effects of Age and BMI group on RT. Although neither Age nor BMI group significantly interacted with cue or target flanker condition, we redid the analysis using Z-transformed RT data^29^ to assess age, BMI, and inflammation effects on visual attention, independently of psychomotor slowing (RT) (see the Statistical methods).

The group mean Z-scores for each network comparison are shown in Table 4. Analyses revealed that alerting (double) cues enhanced performance relative to the no cue condition more in young (*M* = 0.14, s.e. = 0.17) than in old (*M* = −0.40, s.e. = 0.15) adults (*F*(1, 79) = 5.73, *p* = 0.019, η_p_^2^ = 0.07). Young adults were more distracted by incongruent trials (*M* = 2.30, s.e. = 0.15) compared to old adults (*M* = 1.80, s.e. = 0.14; *F*(1, 79) = 6.44, *p* = 0.013, η_p_^2^ = 0.08). No significant age effects were observed for orienting or validity measures (*F*’s < 1). Effects of BMI on all attention measure were non-significant (*F*’s < 1, except for Conflicting, *F* = 1.53, *p* = 0.220). Furthermore, no correlation between any network measure and IL-6 reached significance (*p*’s > 0.5). Hence, no further analyses were performed to test possible mediating effects of IL-6.

**Table 4:** Mean Z-score transformed RT attention network scores (s.e.) shown for each Age and BMI group.

	Alerting	Orienting	Conflicting	Validity
Young Low BMI	0.09 (0.24)	0.76 (0.21)	2.31 (0.21)	1.63 (0.28)
Young High BMI	0.24 (0.24)	1.01 (0.22)	2.30 (0.21)	1.39 (0.28)
Old Low BMI	−0.50 (0.23)	1.17 (0.21)	1.54 (0.20)	1.27 (0.27)
Old High BMI	−0.30 (0.21)	0.93 (0.19)	2.10 (0.18)	1.35 (0.24)

Bayesian correlation analyses showed moderate evidence for H_0_ for a correlation between IL-6 and Z-score alerting (BF_10_ = 0.16), orienting (BF_10_ = 0.17), executive control (BF_10_ = 0.14) and validity effects (BF_10_ = 0.14). Furthermore, Bayesian ANOVAs showed moderate evidence for the null hypothesis (H_0_) against the alternative hypothesis (H_A_) for Z-scores alerting (BMI BF_10_ = 0.32), orienting (BMI BF_10_ = 0.25; ageing BF_10_ = 0.29), and validity (BMI BF_10_ = 0.24) and anecdotal evidence for the H_0_ for executive control in BMI (BF_10_ = 0.47) and validity effect in ageing (BF_10_ = 0.36). The significant effect between age and alerting was supported by Bayesian ANOVA showing moderate evidence for the H_A_ (BF_10_ = 3.10). Anecdotal evidence was found for the age effect on executive control (BF_10_ = 2.42).

## Discussion

The current study examined the relative contributions of inflammation, body weight, and ageing to psychomotor speed and visual attentional processing. As inflammation has been implicated as a putative mechanism for both ageing and BMI associated cognitive decline, the current analyses examined whether these associations, if present, were mediated by inflammation. Although our results confirm earlier findings showing that age and BMI are each associated with psychomotor slowing and inflammation, we show here, for the first time, that inflammation fully mediates BMI-related psychomotor slowing (regardless of age) and that inflammation only partially mediates age-related psychomotor slowing (regardless of body weight). These results partly explain why studies show conflicting results between BMI and psychomotor speed (e.g.^40,42^), since it is not BMI but rather the level of inflammation that is predictive of psychomotor speed. Our results further show that inflammation partially accounts for age-related psychomotor slowing. With regard to visual attention processes, our results show reduced performance benefits of alerting cues (“alerting”) in older adults (after adjusting for psychomotor speed), yet intact, efficient use of spatial information (“orienting”) (e.g.^28,29,63,64^). No relationships were observed between visual attention measures and BMI or inflammation. Together, these results strongly support the contention that inflammation is a significant biological predictor of psychomotor slowing, especially in individuals with high BMI.

A putative pathway by which inflammation might exert cognitive effects is through modulating dopamine pathways^65^. The nigrostriatal dopamine pathway is pivotal in the facilitation of movement, signalling stimulus salience, and motivated behaviours, and it has been demonstrated that inflammation modulates this system^7,65^. Consistent with this speculation, lower levels of striatal dopamine transporter are associated with psychomotor slowing in healthy older adults^66^ and altered function of the dopamine system in individuals with obesity has also been reported^67^. Dopamine function may possibly be altered, directly or indirectly, via microglia. Activated microglia, the brain’s most prominent immune cells, have the capacity to significantly interfere with synaptic turnover, architecture, and function^68^. The substania nigra has a very high density of microglia^69–71^ and activating nigral microglia, for example via local injection of lipopolysaccharides, results in permanent and selective depletion of nigral dopamine^72^. Further indirect support for a role of activated microglia in inflammation-associated psychomotor slowing in humans is the finding that induced acute inflammation in humans, via injection of typhoid vaccination, altered the functional integrity of the microglial-rich substania nigra and was accompanied by psychomotor slowing^7^. Furthermore, in ageing and obesity as well as in certain neurodegenerative conditions, e.g., Alzheimer’s and Parkinson’s disease, microglia shift from a state of relative quiescence to one of activation^73,74^. This results in an enhanced inflammatory response that in turn promotes neural damage and leads to cognitive degradation^4,75^. It is tempting to speculate that disturbance of these functions within brain regions that are involved in psychomotor speed may represent an etiological factor in the onset or progression of psychomotor slowing in inflammatory states. However, considering that age-related psychomotor slowing is not fully explained by elevated inflammation, other mechanisms that are not directly related to inflammation, such as synaptic alterations, white matter lesions, oxidative damage, hormonal changes, and neurochemical changes (e.g.^76,77^) may contribute to psychomotor slowing in advanced age. Moreover, it remains to be determined how peripherally produced inflammatory mediators communicate with the brain to exert its effects on these aforementioned structures and systems. Candidate mechanisms via which cytokines can access the brain include dysfunction of the blood brain barrier (BBB), leaky sections in the circumventricular organs, and retrograde transport via afferent nerves such as the vagus nerve (reviewed in^78^). The current findings are in support of the proposal that reducing inflammation may be useful for mitigating age-related cognitive impairment^79^, and, as suggested by the current data, this benefit may be particularly relevant to individuals with a high BMI. Since psychomotor demands underlie the performance of many routine activities, such as using motorised vehicles, counting out change, and social discourse, means to improve psychomotor speed or delay its age-related degradation are critically. Factors that may partially ameliorate inflammation include modifiable health behaviours such as body weight reduction and improvement of lifestyle (e.g., better diet, more physical activity)^80,81^. Lifestyle adjustments that decrease inflammation may improve cognitive and emotional processes that in turn enhance the ability to engage in and adhere to such lifestyle changes. Not only might this further support a positive cycle of improved health behaviours, such lifestyle adjustments are also relevant to healthy ageing, by reducing risk of diabetes, cardiovascular diseases, and depression^82,83^.

Despite the fact that being overweight or obese is recognised as a major risk factor in the development of health issues and cognitive decline, there is an ongoing debate about possible protective effects of being overweight or obese in old age, referred to as the “obesity paradox”. The obesity paradox has mostly been described in terms of advantageous effects of being overweight or obese for chronic diseases and health outcomes such as coronary heart disease, stroke, high blood pressure, and osteoporosis^84,85^, and has more recently been extended to cognitive processes. The literature on the obesity paradox for cognition is less straightforward compared to that for cardiovascular-associated conditions. Whereas some studies report that higher BMI in midlife is associated with greater cognitive decline in late life^13,86,87^, others report that high mid-life BMI is predictive of better cognitive performance in late life (e.g.^51,88^,) or is non-predictive^43^. These contrasting observations expose the need for greater research in this area and for the development of clarity on mechanisms by which high mid-life BMI might affect central nervous system ageing^89^. Although not longitudinal in design, the current study points towards negative cognitive effects of high BMI-associated inflammation across adulthood, thus arguing against the possibility of cognitive benefits bestowed via the obesity paradox.

The present study has several limitations that warrant consideration. First, the data is correlational in nature. Experimental studies that test whether lowering inflammation, for example through reducing fat mass, restores psychomotor speed may be able to assess a causal role of inflammation and specific mediators. Likewise, it remains to be determined whether chronic inflammation results in permanent disruption of psychomotor functions or whether effects can be restored when inflammation resolves. Lastly, IL-6 was used as a generic index of inflammation but no causal assumptions about the role of IL-6 in the observed effects can be made. Future studies may perhaps incorporate additional markers that may have a proximal role in the effects observed here. At current it is not known which these might be.

In sum, the present study showed that individual differences in psychomotor speed can be accounted for by elevated inflammation linked to being either excessively overweight or older, with effects of each condition being additive, not interactive. Further research focusing on central pathways may help enhance mechanistic understanding of inflammatory effects on cognition. This and other experimental work could help more firmly establish a causal role of inflammation in determining psychomotor speed and reveal whether periods of chronic inflammation result in temporary versus permanent psychomotor slowing.

## Method

### Participants

Eighty-nine participants with a BMI (weight(kg)/height(m)^2^) between 17 and 25 (low BMI) or greater than 27 (high BMI) and aged between 21 and 35 years (young) or between 63 and 80 years (old) were recruited through a database held by the University of Birmingham and via (online) advertisements. Individuals who reported a history of gastric banding, eating disorders, neurological or inflammatory disorders (e.g., rheumatoid arthritis, inflammatory bowel disease, multiple sclerosis, periodontitis) or use of anti-depressant, anti-histamine, or anti-inflammatory (e.g., antibiotics) medication during the past 7 days were excluded. Participants reported normal or corrected-to-normal vision and stable body weight for at least six months (i.e., fluctuations <7.5 kg for individuals with high BMI, <5 kg for low BMI individuals). All data from 6 individuals were excluded from all analyses as their overall behavioural accuracy was on worse than 2.5 s.d.’s below the group average (*N* = 2) or because their overall RT was identified by Cook’s distance as being influential (*N* = 4). Table 1 shows descriptive statistics of the remaining participants’ characteristics. Power analysis calculated using a power of 0.8 and an alpha of 0.05 suggests that a total sample size of 74 is adequate to detect medium to large effect sizes (d = 0.6)^32,39^. The study was conducted according to the guidelines laid down in the Declaration of Helsinki and all procedures were approved by the University of Birmingham Research Ethics Committee.

### Procedures

Test sessions started between 8:30 and 15:30 hours. Start times of test sessions were matched across groups to control for minor diurnal variations in IL-6^90^. However, such variations were not observed in the current study (Spearman’s correlation of IL-6 and time of day (*r*_*S*_(81) = 0.044, *p* = 0.696). Participants were instructed to have breakfast/lunch as usual but avoid consumption of high-fat products (e.g., bacon, fries), because these foods may induce a short-lived inflammatory response^91^, and to refrain from eating, drinking (except for water), and smoking for 1 hour before the start of the test session. Participants were also asked not to engage in strenuous physical exercise or consume alcohol within 12 hours before the test session, and reschedule their appointment if they had suspected infection symptoms on the day of testing. On arrival, written informed consent was obtained and it was verified via self-report whether all had complied with instructions. A blood sample was taken by venipuncture and questionnaires and cognitive tests including the ANT were completed (see further below). Other tests included measures of memory, reward-based learning, and emotion recognition (results not reported here). Lastly, a measure of height and body composition was taken.

## Materials

Questionnaires were completed on a touch screen tablet. A desktop running PsychoPy v1.83.01^92^ was used to record data and present cognitive tests including the ANT.

### Attention network test

#### Stimuli

A fixation cross (1° of visual angle in diameter) was continuously present at the screen’s centre. Warning cues comprised one or two asterisks, each 0.6° in diameter, that appeared either 2.3° left and/or right of the central fixation cross. The test display comprised of a vertical row of five arrows centred horizontally within the screen and appearing either 2.3° left or right of the fixation. The entire row of arrows subtended 3.3° of visual angle; each individual arrow was identical in size (0.6° tall × 1.7° wide) with the space between each being 0.1°. Each arrow could point up or down. In all conditions, the target stimulus was the middle arrow. The two arrows appearing above and below the target (flankers; four in total) always had the same orientation but this could differ from the orientation of the target.

#### Procedure

Each trial began with one of four different warning cues (asterisks; (1) no cue, (2) double cue, (3) central alerting cue, (4) spatial cue), each lasting 100 ms. Regardless of warning cue condition, the cue’s offset preceded the target display by a variable and randomly determined interval of 500‒1000 ms. This display comprised the target and flanker arrows; it disappeared immediately upon response or within 2000 ms. The participant’s task was to indicate the direction of the arrow in the centre of the target display by pressing a corresponding key on the keyboard using the index and middle finger of the dominant hand as quickly and accurately as possible. Response time (RT) was defined as the interval between the onset of the arrow display and the associated keyboard response (in ms). There were 50 trials per participant per warning cue condition; half of all trials per warning cue condition had congruent flankers and half had incongruent flankers. For the spatial cue condition, 80% of trial were valid, 20% invalid.

Every combination of target orientation (up/down) and location (left/right) and distractor compatibility (congruent, incongruent) was equally likely for each warning cue condition. The order of all the various trial types was pseudo-randomly determined for each participant. Participants performed one practice block of 15 trials followed by one experimental block of 200 trials.

#### Blood sampling

Blood was collected into one 6 ml vacutainer containing EDTA as anticoagulant (Becton Dickinson Diagnostics, Oxford, United Kingdom). Samples were immediately centrifuged at 1500 x g for 10 min at 4 **°**C and plasma was aliquoted and stored at −80 **°**C for later assessment of IL-6, a generic marker of systemic inflammation. Plasma level of IL-6 was measured in duplicate using high-sensitivity enzyme-linked immunosorbent assay (ELISA) (Quantikine HS Human IL-6 ELISA, R&D Systems, UK) in accordance with the manufacturer’s instructions. In brief, standards and diluted experimental samples were transferred to a 96-well microplate pre-coated with a monoclonal antibody specific for human IL-6. The IL-6 present was bound by the immobilized antibody. After washing away the unbound substances, the enzyme-linked polyclonal antibody was added to the wells. Following a wash to remove any unbound antibody enzyme reagent, the substrate solution (lyophilized NADPH with stabilizers) was added to the wells. Upon incubation with the experimental samples for 120 min, the amplifier solution (lyophilized amplifier enzymes with stabilizers) was added to the wells for visualization. Readings were subsequently performed using a microplate reader. Data were expressed as pg/mL. The limit of detection of these assays was 0.11 pg/mL, with intra- and interassay coefficients of variation (CVs) of 0.7‒11.6%. IL-6 was used as the primary generic index of inflammation and no causal assumptions about the role of IL-6 in the observed effects are made. No other inflammatory markers were assessed.

#### Questionnaires and anthropomorphic measures

Participants completed health and demographic questionnaires for two reasons. Firstly, to describe the groups and secondly, to adjust for health and demographic factors previously shown to be associated with inflammation, i.e., illness symptoms (Self-Administered Comorbidity Questionnaire (SCQ)^93^, depression, anxiety and stress (Depression Anxiety and Stress-21 (DASS-21)^94^), loneliness (UCLA Loneliness Scale^95^) and demographic variables, i.e., age, education level (low-, middle-, high-educated), perceived health status (rated from ‘1 very poor’ to ‘10 excellent’), alcohol intake (units per week), and smoking status (never-, ex-, current-smoker).

### Statistical analysis

#### RT data

As is conventional (e.g.^21^), RTs were excluded from the statistical analyses if the response was incorrect or too fast (RT < 150 ms), accounting for 3.6% of the data. Also excluded were data from participants who disproportionally bias mean estimates (*N* = 4; using Cook’s distance, 2 young high BMI, 1 old low BMI, 1 old high BMI) and those who had accuracy scores 2.5 s.d.’s lower than the mean (*N* = 2; 2 young high BMI), indicative of poor engagement or inadequate understanding of the task. Individual condition mean RTs and accuracy scores were then subjected to a mixed design repeated measures ANOVA using cue condition (no, double, valid, invalid, and centre cue) and target flanker (congruent and incongruent) as within-subjects factor and Age group and BMI group as between-subjects factors. Psychomotor speed (overall RT) data were then analysed using ANCOVA with age group and BMI group as between-subjects factors and mutual adjustment for age and BMI was applied (i.e., BMI adjusted for age, and age adjusted for BMI). For these analyses and all subsequent analyses where appropriate, Levene’s test of Equality of Variances and Mauchly’s Test of Sphericity were used to test for assumption violations; adjustments were made as needed using the Greenhouse-Geisser correction. Spearman correlations were used to test relationships between age, BMI, IL-6 and RT. Alpha values were set at 0.05 throughout. All analyses were conducted using SPSS v.24.0 (IBM-SPSS Inc., Chicago, IL, USA).

To assess effects of age, BMI and inflammation on attention independent of psychomotor slowing, RT cue and target flanker conditions were *z*-score transformed by taking the individual’s mean score for the condition of interest (e.g., RT double cue trials), subtracting his/her grand average (of all cue and target flanker conditions), and dividing this difference by the standard deviation associated with his/her grand average.

In addition to traditional null hypothesis significance testing, Bayes Factors were calculated for non-significant effects via Bayesian ANOVA using default prior probabilities in JASP version 0.9. Non-significant effects can be the result of absence of differences or because of a lack of statistical power to detect differences. Bayesian analyses can be used to distinguish between these two options^96^. Bayes factors provide relative evidence of both null (H_0_) and alternative hypotheses (H_A_), compared to the conclusions about the null hypothesis proffered by traditional null hypothesis significance testing. To allow for clear interpretation, the approximate classification scheme of^97^ was used which states that an estimated Bayes Factor (BF_10_; H_0_/H_A_) value <1 supports evidence in favour of the H_0_. For example, a BF_10_ of 0.25 indicates that the H_0_ is 4 times (1:0.25) more likely than the H_A_. Values close to 1 are not informative and a BF_10_ between 1 and 3 provides anecdotal evidence for the H_A_. A BF_10_ between 1 and 0.33 provides anecdotal evidence for the H_0_ (e.g., 1:3 probability in favour of H_0_) and a BF_10_ between 0.33 and 0.10 provides moderate evidence for H_0_. A BF_10_ < 0.10 provides strong evidence for H_0_.

#### Mediation effects

PROCESS software^98^ was used to test mediation effects of inflammation (Model 4 with 5000 bootstrap samples). Cook’s distance was used to identify influential data points, which disproportionally bias mean estimates (*N* = 4). Sex, education level (low-, middle-, high-educated), perceived health status (self-report on a scale from 1–10), depression, anxiety, stress (DASS-21), illness symptoms (SCQ), smoking status (never-, ex-, current-smoker) and alcohol intake (units per week) were included as covariates and yielded virtually identical results. Variables were Z-transformed before analysis yielding standardised regression coefficients.

#### IL-6 analysis

A log_10_ transformation was applied to IL-6 data due to the skewed distribution of raw values; correlational and partial correlation analyses were used to adjust for effects of other variables (for example, when assessing the relation between BMI and inflammation, we adjusted for age). Missing values (*N* = 5, 4 = young high BMI, 1 = old high BMI) and extreme plasma IL-6 concentrations (>6 pg/ml) (*N* = 4, 3 = old low BMI, 1 = young high BMI) were replaced by group mean values. Repeating the main IL-6 analyses (Spearman correlations and ANOVAs) without replacement of IL-6 values produced similar results (see Supplementary information Table S3)^99^.

## Supplementary information

Supplementary Information

## Data Availability

The dataset of this article is accessible on reasonable request from the corresponding author.
