# Supplementary material for: Inflammation Mediates Body Weight and Ageing Effects on Psychomotor Slowing
Source: Sci Rep. 2019 Oct 31;9:15727. doi: 10.1038/s41598-019-52062-3 (PMC6823347; doi:10.1038/s41598-019-52062-3)
Supplement: Supplementary file 1 — Supplementary Information [file 41598_2019_52062_MOESM1_ESM.docx]

## Inflammation Mediates Body Weight and Ageing Effects on Psychomotor Slowing

Leonie JT Balter^A, C^*, Suzanne Higgs^A^ , Sarah Aldred ^B^, Jos A Bosch ^C⊥^, & Jane E Raymond ^A⊥^

^A^School of Psychology, University of Birmingham, Birmingham, B15 2TT, UK

^B^School of Sport, Exercise, and Rehabilitation Sciences, University of Birmingham, Birmingham, B15 2TT, UK

^C^Psychology Department, Clinical Psychology, University of Amsterdam, Amsterdam, 1018 WT, NL

^⊥^Shared senior authorship

*Corresponding Author:

Email: [l.j.t.balter@uva.nl](mailto:l.j.t.balter@uva.nl)

##### SUPPLEMENTARY INFORMATION

**Table S1.**

Results from hierarchical regression analysis showing the mediation effect of

IL-6 on the relationship between BMI group and response time (RT). Step 1 involved entering BMI group, followed by IL-6 in step 2 and demographic and health variables in step 3. BMI group was coded as low (1) and high (2) BMI, Sex was coded as male (1) and female (2), and Education level was coded as low (1), middle (2), and high (3) education. Statistical significance is indicated as follows; * *p* < .05, ** *p* < .01, *** *p* < .001

|  | Step 1  R^2^ = .069*  ΔF = 5.845* | | | Step 2  R^2^ = .184***  ΔF = 10.983** | | | Step 3  R^2^ = .346**  ΔF = 1.689 | | |
| --- | --- | --- | --- | --- | --- | --- | --- | --- | --- |
|  | β | t | *p* | β | t | *p* | β | t | *p* |
| BMI Group | 0.262 | 2.418 | .018 | 0.006 | 0.049 | .961 | -0.018 | -0.134 | .894 |
| IL-6 |  |  |  | 0.425 | 3.314 | .001 | 0.322 | 2.450 | .017 |
| Time of day tested |  |  |  |  |  |  | 0.135 | 1.301 | .198 |
| Sex |  |  |  |  |  |  | -0.097 | -0.830 | .410 |
| Education level |  |  |  |  |  |  | -0.148 | -1.220 | .227 |
| Perceived health status |  |  |  |  |  |  | -0.005 | -0.040 | .968 |
| Depression |  |  |  |  |  |  | 0.053 | 0.311 | .757 |
| Anxiety |  |  |  |  |  |  | -0.192 | -1.348 | .182 |
| Stress |  |  |  |  |  |  | -0.198 | -1.146 | .256 |
| Illness symptoms |  |  |  |  |  |  | 0.199 | 1.744 | .086 |
| Smoking status |  |  |  |  |  |  | -0.083 | -0.675 | .502 |
| Alcohol intake |  |  |  |  |  |  | 0.046 | 0.368 | .714 |

**Table S2.**

Results from hierarchical regression analysis showing the mediation effect of IL-6 on the relationship between Age group and response time (RT). Step 1 involved entering Age group, followed by IL-6 in step 2 and demographic and health variables in step 3. Age group was coded as young (1) and old (2), Sex was coded as male (1) and female (2), Education level was coded as low (1), middle (2) and high (3) education. Statistical significance is indicated as follows; * *p* < .05, ** *p* < .01, *** *p* < .001

|  | Model 1  R^2^ = .451***  ΔF = 64.940*** | | | Model 2  R^2^ = .529***  ΔF = 12.957** | | | Model 3  R^2^ = .592***  ΔF = 1.043 | | |
| --- | --- | --- | --- | --- | --- | --- | --- | --- | --- |
|  | β | t | *p* | β | t | *p* | β | t | *p* |
| Age Group | 0.672 | 8.059 | <.001 | 0.605 | 7.568 | <.001 | 0.705 | 6.401 | <.001 |
| IL-6 |  |  |  | 0.288 | 3.600 | .001 | 0.264 | 2.991 | .004 |
| Time of day tested |  |  |  |  |  |  | 0.135 | 1.654 | .103 |
| Sex |  |  |  |  |  |  | -0.061 | -0.658 | .513 |
| Education level |  |  |  |  |  |  | -0.133 | -1.416 | .161 |
| Perceived health status |  |  |  |  |  |  | -0.034 | -0.387 | .700 |
| Depression |  |  |  |  |  |  | 0.109 | 0.824 | .413 |
| Anxiety |  |  |  |  |  |  | 0.007 | 0.058 | .954 |
| Stress |  |  |  |  |  |  | -0.115 | -0.840 | .404 |
| Illness symptoms |  |  |  |  |  |  | -0.082 | -0.826 | .412 |
| Smoking status |  |  |  |  |  |  | -0.084 | -0.871 | .387 |
| Alcohol intake |  |  |  |  |  |  | -0.079 | -0.783 | .437 |

**Table S3.**

Spearman correlations and ANOVAs for the relationships between IL-6 and age, IL-6 and BMI, and IL-6 and RT, either with (N = 83) or without (N = 74) replacement of IL-6 values for group mean IL-6 values of individuals with missing or extreme IL-6 concentrations. Statistical significance is indicated as follows; * *p* < .05, ** *p* < .01, *** *p* < .001

|  | *N* = 83  (with replacement) | | | *N* = 74  (without replacement) | | |
| --- | --- | --- | --- | --- | --- | --- |
|  | *r_s_* | *p* | *F* | *r_s_* | *p* | *F* |
| Age | .320 | .003 | 4.55* | .416 | <.001 | 4.85* |
| BMI | .655 | <.001 | 49.25*** | .638 | <.001 | 33.97*** |
| RT | .430 | <.001 |  | .489 | <.001 |  |
